# Supplementary material for: Daily steps are a predictor of, but perhaps not a modifiable risk factor for Parkinson’s Disease: findings from the UK Biobank
Source: medRxiv. 2024 Aug 14:2024.08.13.24311539. Preprint. [Version 1] doi: 10.1101/2024.08.13.24311539 (PMC11451817; doi:10.1101/2024.08.13.24311539)
Supplement: Supplement 1 [file media-1.pdf]

Supplementary materials

Tables

Supplementary Table 1: Description of datasets used in this analysis.

**Characteristic**	**OxWalk study**	**UK Biobank Physical Activity Cohort**
No. of participants	39	~ 100,000
Sensor	Axivity AX3	Axivity AX3
Raw sampling rate	100Hz	100Hz
Body location	Dominant wrist	Dominant wrist
Activity protocol	Unscripted free-living	Unscripted free-living
Measurement window, per participant	1 hour	7 days
Ground truth capture	Waist worn, foot facing, video camera	None
Participant Age	38.5 (SD 14.0)<br>Range: 19 - 81	62.4 (SD 7.8)<br>Range: 43 - 79
Participant Sex	19 Female, 20 Male	~56,000 Female,<br>~44,000 Male

Supplementary Table 2: Definition of variables from the UK Biobank data

Characteristic	Source	Notes	UK Biobank field	Coding Notes
**OUTCOME**				
**Age at first PD event**	Hospital episode statistics (HES) records	First ICD-10 code (G20) in HES data.	Derived from 2000	
**Age loss-to-follow up**	Death Registry		Derived from 100093	
**EXPOSURE**				
**Median Daily Step count**	Accelerometry	Derived using the "stepcount" tool, version 2.1.5.<br>Details can be found at:<br>https://github.com/OxWearables/stepcount		Reported either as divided into tertiles:<br><7,560, 7,560-10,646, 10,646+<br>Or as per-1000 daily steps
**EXCLUSION VARIABLES**				
**Prior Parkinsonism**	HES records	PD-related ICD-10 codes in hospital records before accelerometry wear date:<br>G20-26, G35, G47	Derived from 2000	
	Baseline	PD-related self-reported disease	Derived from 20002	
	Algorithmically derived first instance of PD (G20)	Date G20 first reported (Parkinson's disease) prior to the accelerometry wear date	Derived from 131022	
**ADJUSTMENT VARIABLES**				
**Age**	Baseline	Attained age was the underlying timescale in survival analyses; participants entered the study at the end of accelerometer wear.	Derived from 90011, 34, 52	

**Sex**	Baseline		31	Female, Male
**Ethnicity**	Baseline		Derived from 21000	White, non-white
**Townsend Deprivation Index**	Baseline	Townsend Deprivation Index of address at time of UKB baseline assessment. UK population quintiles: linked here	Derived from 22189	Quintiles of UK Population
**UK recruitment centre geographic region**	Baseline		Derived from 54	London, West Midlands, Yorkshire, northeast, northwest, southeast, southwest England, Scotland, Wales
**Education/Qualifications**	Baseline	Selected as highest educational achievement. Labels are ordered by highest to lowest value. ISCED UK Biobank Paper	Derived from 6138	College/University degree, NVQ/HND/HNC or equivalent, Other professional qualification, A levels/AS levels or equivalent, GCSEs/CSEs/O levels or equivalent, No qualification
**Employment status**	Baseline		Derived from 6142, 20119	Employed, Not employed
**Smoking status**	Baseline		Derived from 20116	Never, previous, current
**Alcohol consumption**	Baseline		Derived from 1558	Never, <3 times per week, ≥3 times per week
**Coffee intake**	Baseline		Derived from 1498	Non-drinker, Drinker - up to 2 cups, Drinker - more than 2 cups
**ADJUSTMENT VARIABLES – SENSITIVITY ANALYSIS**				
**Body mass index**	Baseline		Derived from 21001	
**Average sleep duration**	Accelerometry		Derived from 40046	
**Type 2 Diabetes**	HES records	ICD-10 code (E11) in HES data prior to end of accelerometer wear.	Derived from 2000	
	Baseline	Type 2 Diabetes self-reported disease at baseline	Derived from 20002, 2443	

**Depression**	HES records	ICD-10 code (F32, 33) in HES data prior to end of accelerometer wear.	Derived from 2000	
	Baseline	Depression self-reported disease at baseline	Derived from 20002, 2090, 2100	
**Constipation**	HES records	ICD10 code (K59.0) in HES data prior to accelerometer wear	Derived from 2000	
	Baseline	Use of laxatives in self-reported medication use at baseline	Derived from 6154	
**Bladder dysfunction**	HES records	ICD10 code (N31) in HES data prior to accelerometer wear	Derived from 2000	
	Baseline	Incontinence self-reported disease at baseline	Derived from 20002	

**Neurological conditions**	HES records	Any of the following ICD10 codes in HES data prior to accelerometer wear:<br>A80, A80.0, A80.1, A80.2, A80.3, A80.4, A80.8, A80.9, D32, D36, E10.4, E11.4, E12.4, E13.4, E14.4, F00, F00.0, F00.1, F00.2, F00.9, F01, F01.0, F01.1, F01.2, F01.3, F01.8, F01.9, F02, F02.0, F02.1, F02.2, F02.3, F02.4, F02.8, F03, F05, F05.1, G00, G01, G02, G03, G04, G06, G07, G12, G25.0, G30, G30.0, G30.1, G30.8, G30.9, G31, G31.1, G32, G35, G36, G37, G40, G43, G44, G50, G50.0, G50.1, G50.8, G50.9, G51, G51.0, G52, G53, G57, G60, G61, G61.0, G62, G70.0, G72, G80, G81, G82, G82.2, G82.3, G82.4, G83, G83.0, G83.1, G83.2, G83.3, G83.4, G83.8, G83.9, G84, G90, G91, G92, G93, G93.0, G93.1, G93.2, G93.3, G93.4, G93.5, G93.6, G93.7, G93.8, G93.9, G94, G95, G96, G96.0, G96.1, G96.8, G96.9, G97, G98, G99, H53, H81, H81.0, H81.1, H81.2, H81.3, H81.4, H81.8, H81.9, H83, H90, H91, H91.0, H91.1, H91.2, H91.3, H91.8, H91.9, H93, H93.0, H93.1, H93.2, H93.3, H93.8, H93.9, I67, I67.0, I67.1, I67.2, I67.3, I67.4, I67.5, I67.6, I67.7, I67.8, I67.9, K56, M79, M79.0, M79.1, M79.2, M79.3, M79.4, M79.6, M79.7, M79.8, M79.9, N08, N08.0,	Derived from 2000	

		N08.8, N08.9, N13, N13.0, N13.1, N13.2, N13.3, N13.4, N13.5, N13.6, N13.7, N13.8, N13.9, Q05, R29.0, R41.81, S0[0-9], S14, S14.0, S24, S24.0, S24.1, S34, S34.0, S34.1, S34.3, S44, S54, S64, S74, S84, S94, T04, T06, T07, T14, T24, T84, T90, T90.3, T90.4, T90.5, T90.8, T90.9, T91, T91.3		

	Baseline	Any of the following self-reported diseases at baseline:<br>infection of nervous system, brain abscess/intracranial abscess, encephalitis, meningitis, spinal abscess, cranial nerve problem/palsy, bell's palsy/facial nerve palsy, trigemminal neuralgia, spinal cord disorder, paraplegia, spina bifida, peripheral nerve disorder, peripheral neuropathy, acute infective polyneuritis/guillain-barre syndrome, trapped nerve/compressed nerve, diabetic neuropathy/ulcers, chronic/degenerative neurological problem, motor neurone disease, myasthenia gravis, multiple sclerosis, parkinsons, dementia, alzheimers, cognitive impairment, other demyelinating disease, epilepsy, migraine, cerebral palsy, other neurological problem, headaches, benign / essential tremor, polio / poliomyelitis, meningioma / benign meningeal tumour, benign neuroma, neurological injury/trauma, head injury, spinal injury, peripheral nerve injury	Derived from 20002	
**DESCRIPTIVE VARIABLES – DESCRIPTIVE TABLES**				
**Wear season**			Derived from 90001	Winter, Spring, Summer, Autumn

Supplementary Table 3: Strengthening the Reporting of Observational Studies in Epidemiology (STROBE) guidelines checklist for reporting of findings with this work. Pages with the S prefix correspond to supplementary material, otherwise refer to the main manuscript.

**Section**	**Subsection**	**Summary**	**Code**	**Pages**
Title and Abstract	Title and Abstract	Indicate study's design in title/abstract	1a	1-3
Title and Abstract	Title and Abstract	Provide informative summary of study	1b	2-3
Introduction	Background/rationale	Explain scientific background and rationale	2	4
Introduction	Objectives	State specific objectives and hypotheses	3	4
Methods	Study design	Present key elements of study design	4	4-6
Methods	Setting	Describe setting, locations, and relevant dates	5	4
Methods	Participants	Give eligibility criteria and selection methods	6a	4-5
Methods	Participants	Provide matching criteria for matched studies	6b	N/A
Methods	Variables	Define outcomes, exposures, confounders, and effect modifiers	7	5-6
Methods	Data sources measurement	Give sources and methods of data assessment	8	5-6
Methods	Bias	Describe efforts to address bias	9	6
Methods	Study size	Explain how study size was determined	10	N/A
Methods	Quantitative variables	Explain handling of quantitative variables	11	5
Methods	Statistical methods	Describe all statistical methods used	12a	5
Methods	Statistical methods	Describe methods for examining subgroups and interactions	12b	6
Methods	Statistical methods	Explain how missing data were addressed	12c	5

Methods	Statistical methods	Explain how loss to follow-up was addressed	12d	6
Methods	Statistical methods	Describe any sensitivity analyses	12e	6
Results	Participants	Report numbers at each stage of the study	13a	6
Results	Participants	Give reasons for non-participation	13b	N/A
Results	Participants	Consider use of a flow diagram	13c	16
Results	Descriptive data	Provide characteristics of study participants	14a	6-7
Results	Descriptive data	Indicate number of participants with missing data	14b	16
Results	Descriptive data	Summarise follow-up time	14c	6
Results	Outcome data	Report numbers of outcome events or summary measures	15	6
Results	Main results	Give unadjusted and adjusted estimates with precision	16a	7-8, 17-18, S11
Results	Main results	Report category boundaries when continuous variables were categorized	16b	S3-7
Results	Main results	Translate estimates of relative risk into absolute risk if relevant	16c	N/A
Results	Other analyses	Report other analyses done	17	7-8
Discussion	Key results	Summarise key results with reference to objectives	18	8
Discussion	Limitations	Discuss the limitations of the study	19	9
Discussion	Interpretation	Provide overall interpretation of results	20	8-9
Discussion	Generalisability	Discuss the generalisability of the study results	21	9
Other information	Funding	Give the source of funding and role of funders	22	3

Figures

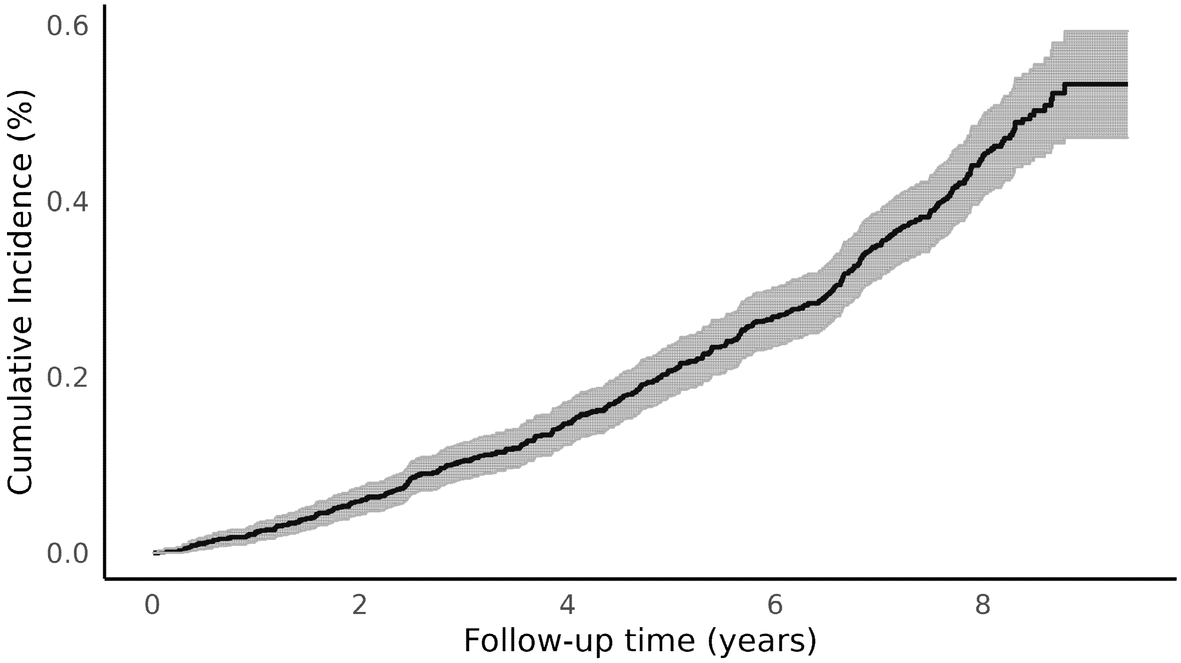

Supplementary Figure 1: Kaplan-Meier curve for cumulative incidence of Parkinson's disease in the UK Biobank physical activity monitoring study population.

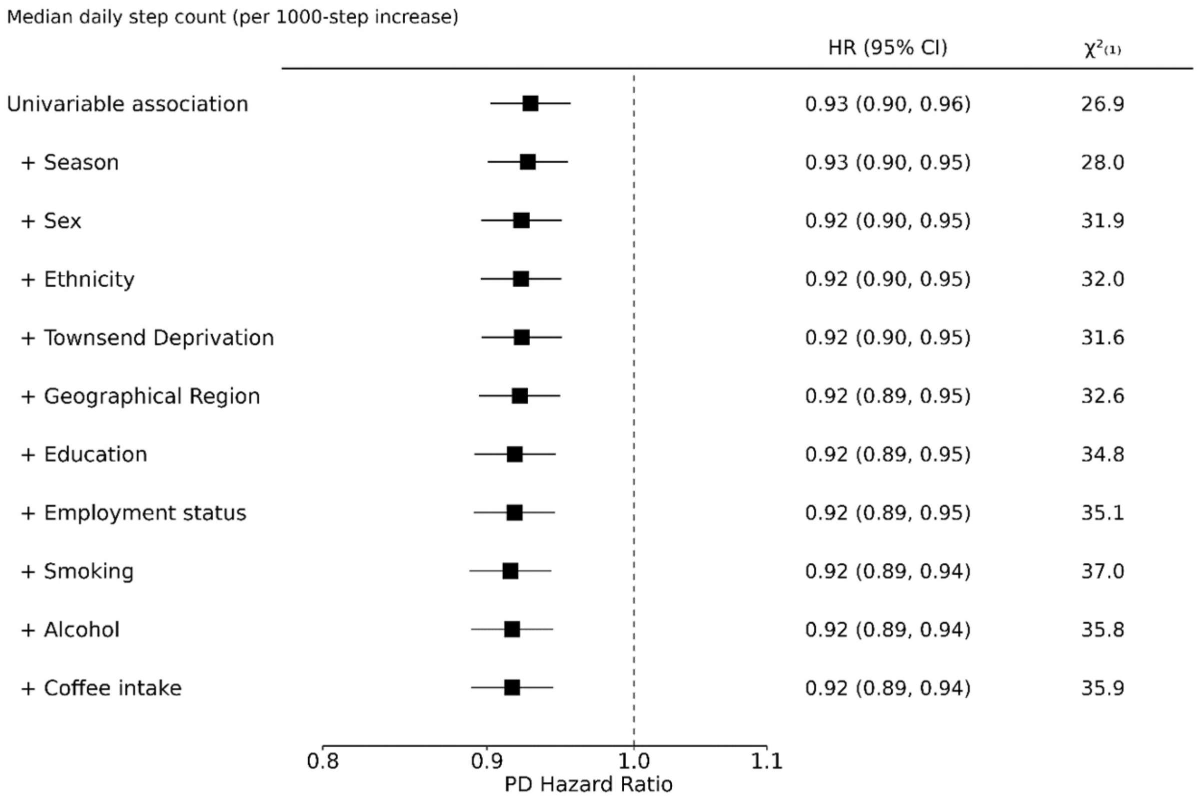

Supplementary Figure 2: The sequential adjustment of the association between a per 1000-step increase in imputed median daily step count and incident Parkinson's disease with age as timescale using all periods of follow-up. HR=Hazard ratio, CI=Confidence interval, $\chi^2_{(1)}$=chi squared using likelihood ratio test for one degree of freedom.

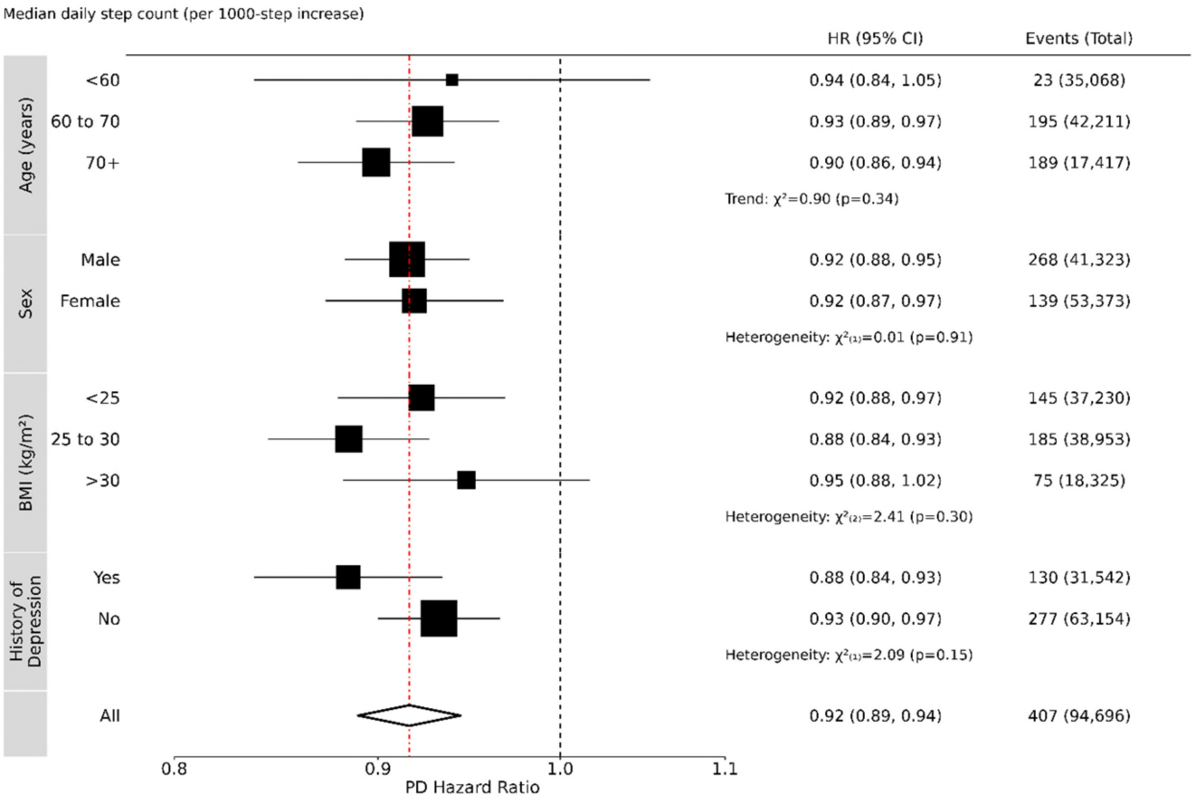

Supplementary Figure 3: The association between a per 1000-step increase in imputed median daily step count and incident Parkinson's disease with age as timescale using all periods of follow-up within various subpopulations. Models are adjusted for season, sex (except for sex-based sub-populations), ethnicity, Townsend deprivation, geographic region, education, employment status, smoking, alcohol and coffee intake. BMI=Body Mass Index, HR=Hazard ratio, CI=Confidence interval.

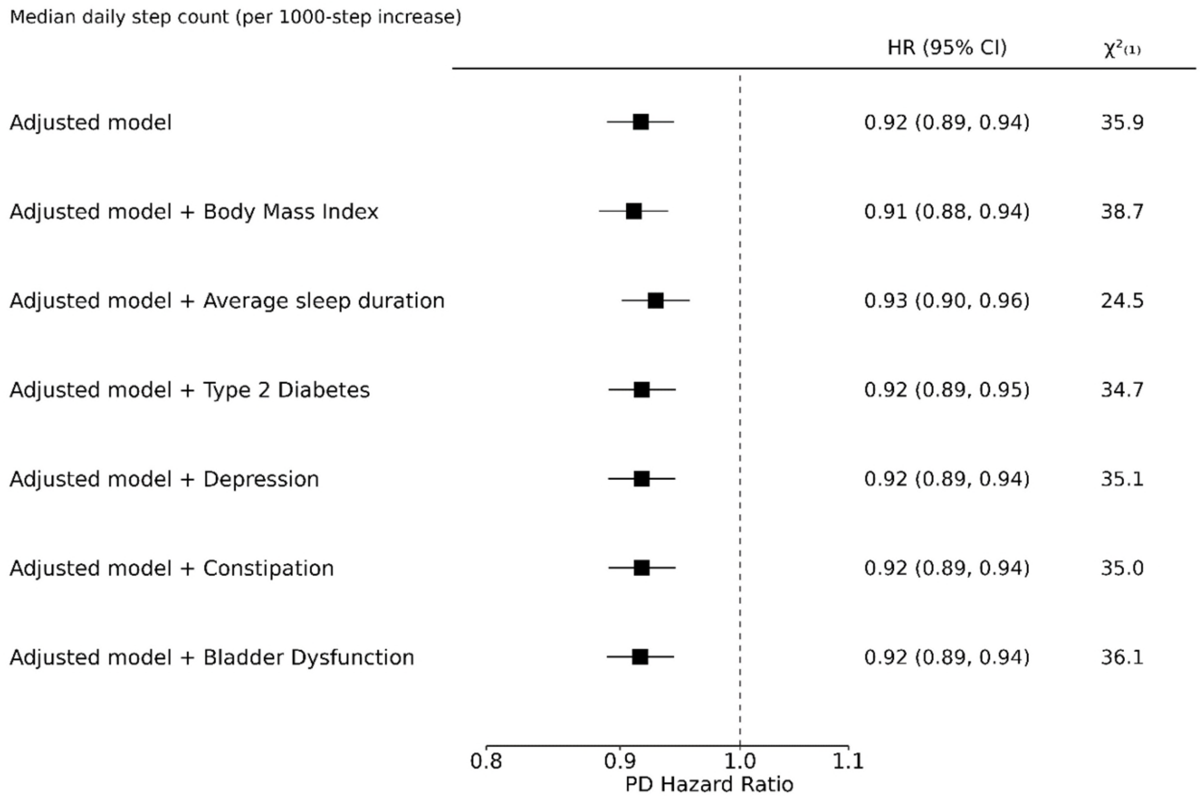

Supplementary Figure 4: The association between a per 1000-step increase in imputed median daily step count and incident Parkinson's disease with age as timescale using all periods of follow-up after adjustment for potential confounding factors. The adjusted model is adjusted for season, sex, ethnicity, Townsend deprivation, geographic region, education, employment status, smoking, alcohol and coffee intake. HR=Hazard ratio, CI=Confidence interval, $\chi^2_{(1)}$=chi squared using likelihood ratio test for one degree of freedom.
